# The effect of different functional appliances on the sagittal pharyngeal airway dimension in skeletal class II: a retrospective study

**DOI:** 10.1038/s41598-024-69717-5

**Published:** 2024-08-21

**Authors:** Dina Elfouly, Emmanuel Jr. Dumu, Ahmed M. Madian, Farah Y. Eid

**Affiliations:** 1 Department of Orthodontics, Faculty of Dentistry, Alexandria University, Champolion street, Azarita, Alexandria, Egypt; 2 Pebs Dental Clinic, Brussels, Belgium

**Keywords:** Class II, Invisalign mandibular advancement, Myobrace, Twinblock, Myofunctional appliance, Sagittal pharyngeal airway dimension, Health care, Medical research

## Abstract

The aim of this study was to compare the changes in the sagittal pharyngeal airway dimension (SPAD) in adolescents with Class II mandibular retrusion treated with Invisalign Mandibular Advancement (IMA), prefabricated Myobrace (MB), and Twin block (TB). For this retrospective study, the pre-treatment and post-treatment lateral cephalograms of 60 patients who underwent myofunctional treatment, using either one of the tested appliances were gathered from the files of treated patients. Changes in the SPAD were measured in each group, and comparisons were carried out between the three study groups. Additionally, sagittal skeletal measurements were carried out. Comparisons of the study variables at T0 and T1 between the three groups were performed using one-way ANOVA, while comparisons of the difference (T1–T0) were performed using Kruskal Wallis test. A significant SPAD increase has been reported using the three tested appliances (p < 0.05), with the least change documented with MB use (p < 0.05). Significant antero-posterior improvements have been found with IMA, MB, and TB with an increase in the SNB°, and a decrease in ANB° and Wits appraisal (p < 0.05). Non-significant FMA° changes have been observed post-treatment in the three test groups (p > 0.05). The IMA, MB, and TB generated significant SPAD and sagittal changes, with both IMA and TB surpassing MB in the airway area improvement post-treatment. Moreover, the three tested Class II functional appliances did not affect the vertical dimension.

## Introduction

Skeletal Class II malocclusion is regarded as one of the most prevailing dentofacial abnormalities, which has been found to affect approximately one-third of the population^1^. It may be characterized by mandibular retrognathism, maxillary prognathism, or a combination of both skeletal deviations^2,3^.

As a potential consequence to mandibular retrognathia, the area between the cervical column and the mandibular corpus is diminished, and the tongue and soft palate are both posteriorly postured; thus, the airway dimensions are eventually narrowed^4^. This in turn explains the reduced pharyngeal airway dimensions reported in Angle Class II division 1 subjects^5,6^. It has been documented that in cases of early skeletal Class II diagnosis, the best treatment option is functional appliances, which promote forward mandibular growth and prevent upper airway collapse during sleep^7–10^.

The Twin Block (TB) appliance is one of the most used removable functional appliances conventionally indicated for correcting a retrognathic mandible in Class II malocclusion^11,12^. It consists of two overlapping plates with inclined acrylic surfaces, that posture the lower jaw in a forward position on closure^13^. TB has also been reported to increase the pharyngeal airway dimensions through the forward movement of the mandible and the hyoid bone^7,14,15^.

Myobrace (MB) is a preformed orthodontic device, designed for the treatment of malocclusions in patients in the late mixed dentition (9–12 years). It attempts to retrain the tongue’s position and enhance the equilibrium of the masticatory and facial muscles. MB pursues three goals, i.e.: mandibular development, dental alignment, in addition to a myofunctional impact. It is constructed using an edge-to-edge incisal relation and consists of a single block that touches both arches. The sole structural difference, in contrast to the other “Trainer System” devices, is an internal additional hard nylon element, called “Inner-Core”, or “Dynamicore”^16^.

In 2017, Align Technology (San Jose, CA, USA) released the Invisalign® Mandibular Advancement (IMA) appliance, which is a functional appliance that integrates both the notions of growth modification and active tooth movement^13^. IMA replicates the mechanism of action of functional appliances through the engagement of inclined planes embedded into buccal precision wings, located between the first molars and premolars when the subject is in occlusion. Align Technology with the IMA appliance, targeted growing patients with mild to severe mandibular retrognathism, in the presence of a permanent or a stable late mixed dentition^13^.

With the pharyngeal size considered a cardinal factor influencing the quality of sleep^17^, the reduced size of the nasopharynx has a negative impact on the pattern of breathing, whether oral or nasal^18^. The competency of the functional appliances in enlarging the airway dimensions has been widely investigated in the literature, with a recent study reporting that both IMA and TB appliances have successfully improved the structural narrowness of the upper airway^15,19^. However, none of the previous studies have contemplated the possible differences in the effects of IMA with MB and TB on the sagittal pharyngeal airway dimension (SPAD) in adolescents.

Thus, the aim of this study was to compare the changes in the SPAD of subjects with Class II mandibular retrusion treated with IMA, MB, and TB appliances. Moreover, sagittal and vertical skeletal changes accompanying each of the tested appliances were assessed. The null hypothesis was that there are no significant differences between the three studied functional devices regarding their influence on the SPAD.

## Methods

### Patient selection

Approval was attained for this retrospective study by the Institutional Review Board of the Faculty of Dentistry, Alexandria University, Alexandria, Egypt (IRB:00010556–IORG:0008839). Manuscript Ethics Committee number 0822-12/2023. Sixty pre- and post-treatment lateral cephalograms of subjects treated at the Orthodontic Department, Alexandria University, Egypt and at Pebs Dental Practice, Brussels, Belgium until October 2023, were checked for qualification by the principal investigator. All the investigative procedures were carried out in agreement with the relevant guidelines and regulations, as stated in the Declaration of Helsinki. Oral approvals and written informed consents were acquired from the patients and/or their legal guardian(s) for the use of their records in this research.

The sample size was based on 95% confidence level to detect differences in airway measurements after treatment with Invisalign mandibular advancement (IMA) and TwinBlock (TB) appliances. Yue et al.^19^ reported mean (SD) change in oropharyngeal airway volume (mm^3^) = 1691.89 (1574.67) and 2194.56 (1437.15), after treatment with IMA and TB appliances, respectively. The calculated mean (SD) difference = 502.67 (1505.91) and 95% confidence interval = − 585.81, 1591.15. Myobrace is assumed to have a similar effect on airway measurements as TB appliance^20^. The sample size was calculated to be 18 patients, increased to 20 to compensate for cases lost to follow up. The total required sample size = number of groups × number per group = 3 × 20 = 60 patients^21^. The employed software for sample size calculation was MedCalc Statistical Software version 19.0.5 (MedCalc Software bvba, Ostend, Belgium; https://www.medcalc.org; 2019).

Inclusion criteria for patient enrolment included: (1) Age ranging from 9 to 12 years, (2) Initial overjet between 5 and 8 mm, (3) skeletal Class II subjects with mandibular retrognathism (ANBº > 4°, SNBº < 78°) that have been treated with IMA, MB, or TB appliances, (4) visible profile enhancement on forward mandibular posturing, (5) cervical vertebral maturational index (CVMI) stage 3, as dictated from lateral cephalograms^22^, (6) availability of pre-treatment (T0) and post-treatment (T1) lateral cephalometric radiographs. In all the included subjects, functional appliance treatment was concluded after achieving Class I molar relationship. As for the exclusion criteria, they involved: (1) prior orthodontic treatment, (2) cleft lip and/or palate patients, (3) subjects with craniofacial anomalies or diagnosed syndromes, (4) history of tonsillectomy or maxillofacial surgery.

A consistent technique has been carried out while taking all the attained lateral cephalograms, where the subjects stood in natural head position^23,24^, as well as natural tongue posture with the teeth in centric occlusion. Instructions were given to stand still, and not to move the head nor swallow in the course of the exposure. Lateral cephalometric X-rays were compared between T0 and T1 for the assessment of SPAD, as well as the vertical and sagittal skeletal alterations in all the intervention groups.

### Grouping

**Figure 1:**
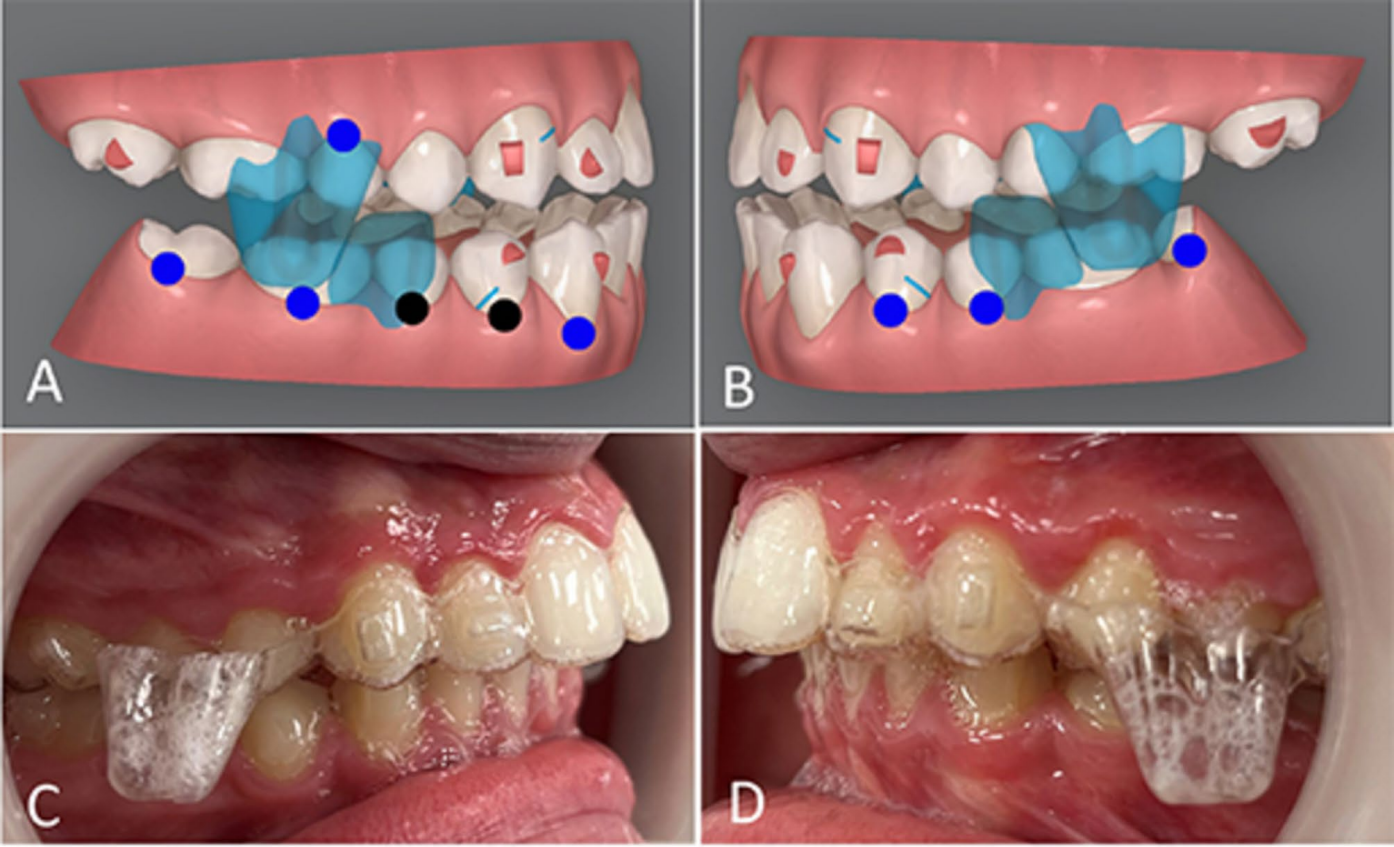
(**A**, **B**) Right and left views of the ClinCheck plan for the IMA. (**C**, **D**) Right and left intra-oral views for the IMA appliance.

**Figure 2:**
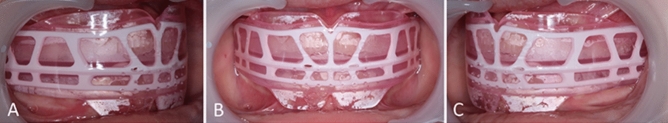
(**A**) Right, (**B**) frontal, and (**C**) left intra-oral views of the MB appliance.

**Figure 3:**
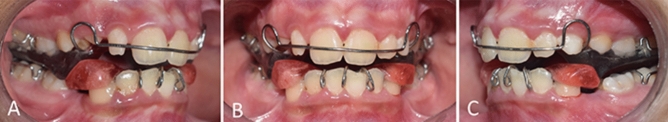
(**A**) Right, (**B**) frontal, and (**C**) left intra-oral views of the TB appliance.

The procured lateral cephalometric records were split into three groups based upon the Class II corrective appliance used; Group A: Invisalign Mandibular Advancement (Align Technology, San Jose, CA, USA) (Fig. 1), Group B: Myobrace (prefabricated functional appliance, Myofunctional Research Co., Australia) (Fig. 2), and Group C: Twinblock (Fig. 3).

### Appliances’ wear protocol

Regarding the TB group, patients were instructed to wear the appliance full time except during meals^25,26^. In the MB group, patients were instructed to wear the appliance for a minimum of 2 h/day, throughout the first week after appliance delivery. By the second week, patients were instructed to use the device for at least eight hours every night. Patients then wore their appliances for a minimum of 1–2 h per day and overnight starting from the conclusion of the first 4 weeks of therapy and continuing throughout the treatment period, as per the manufacturer’s instructions^27^. For the IMA group, patients were required to wear their aligners for at least 22 h/day, which can be removed while eating. They were also notified to replace the aligners on a weekly basis according to their planned set of aligners^19^.

For the three study groups, monthly follow-ups were scheduled until a Class I molar relationship has been achieved. Since patient’s compliance was of prime importance during the treatment period, a follow-up chart was prepared and monitored periodically^28^. Moreover, overjet, overbite, and molar relations were recorded every 12th week. At the end of the treatment, for MB and TB groups, subjects were instructed to wear the appliance as a retainer according to the operator’s preference^28,29^. Regarding the IMA group, they were given Vivera retainers (Align technology, San Jose, CA, USA) after the final set of aligners.

### Outcomes’ assessment

#### Sagittal pharyngeal airway dimension changes

**Figure 4:**
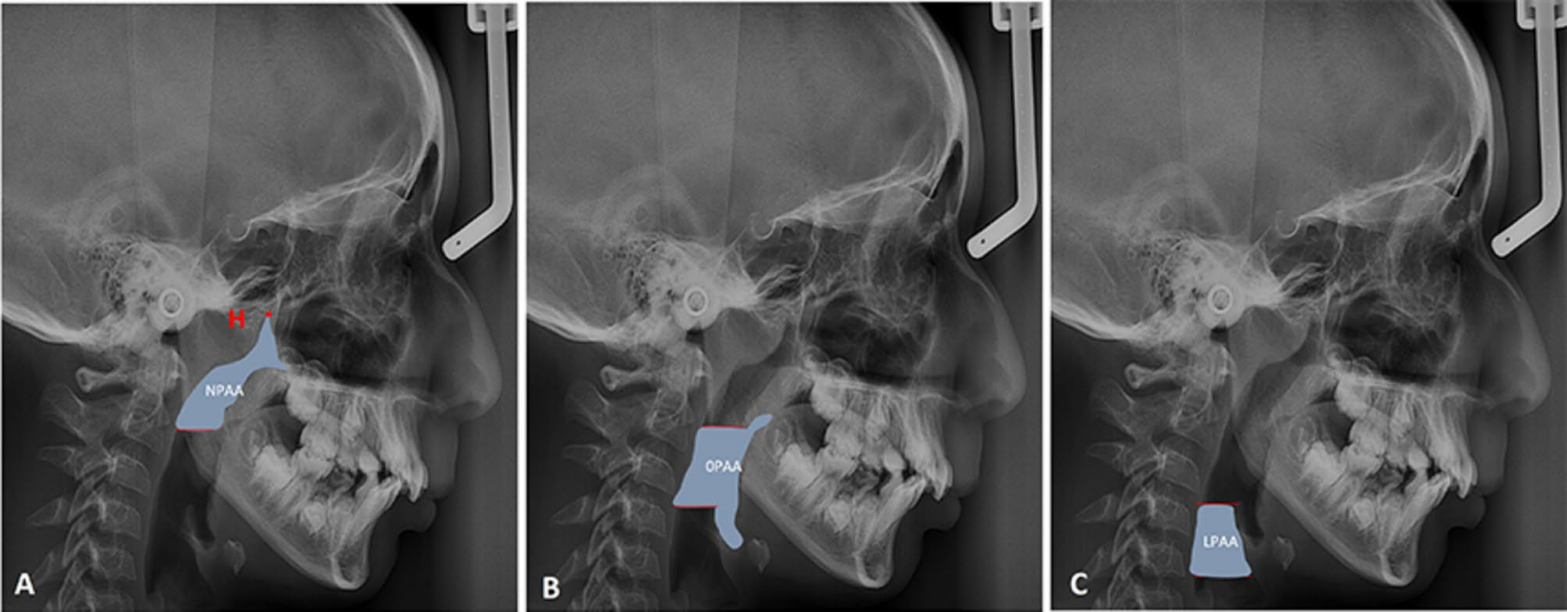
The implemented cephalometric sagittal pharyngeal airway area measurements (H: Harmonium). (**A**) Nasopharyngeal airway area (NPAA), (**B**) Oropharyngeal airway area (OPAA), (**C**) Laryngopharyngeal airway area (LPAA).

Digital tracing was accomplished using Osirix open-source software for the obtained lateral cephalograms^30^, where a myriad of landmarks was identified. The sagittal pharyngeal airway space was separated into the Nasopharyngeal airway area (NPAA), the Oropharyngeal airway area (OPAA), and the Laryngopharyngeal airway area (LPAA)^31^. A line extending from the Harmonium (H) to the posterior nasal spine (PNS) demarcated the upper border of the NPAA. The lower extent of the NPAA was traced by drawing a line at the tip of the soft palate parallel to the Frankfurt Horizontal plane (FH), stretching out to the posterior pharyngeal wall. The OPAA and LPAA were distinguished by a line drawn at the level of the tip of epiglottis, parallel to the FH plane to the posterior wall of the pharynx. The lower border of the LPAA was marked by a line drawn parallel to FH plane, passing through the antero-inferior most point (C5AI) of the fifth cervical vertebra. The area has been measured using the same software in mm^2^ (Fig. 4).

#### Sagittal and vertical skeletal changes

From the acquired pre- and post-treatment lateral cephalograms and with the same software being employed (Osirix open-source software), distinct linear and angular measurements were performed, for appraisal of the resultant changes in both sagittal and vertical planes (Table 1). For sagittal dimension assessment, SNA°, SNB°, and ANB°, as well as Wits appraisal were measured. Regarding the vertical dimension, the Frankfurt-mandibular plane angle (FMA°) was measured for evaluation.

**Table 1:** Skeletal measurements; SNA°, SNB°, ANB°, Wits appraisal, FMA°.

SNA°	Angle between the points Sella (S), Nasion (N) and A point, showing the anteroposterior position of the maxilla relative to the anterior cranial base
SNB°	Angle between points Sella (S), Nasion (N), and B point, describing the anteroposterior position of the mandible relative to the anterior cranial base
ANB°	Angle between points A, Nasion (N) and B point, indicating the skeletal relationship between the maxilla and the mandible
Wits appraisal (mm)	The linear distance between the perpendicular projections of points A and B over the functional occlusal plane
FMA°	Angle between the Mandibular plane and the Frankfort horizontal plane

### Blinding

The researcher was blinded while performing the measurements on the lateral cephalometric radiographs, and the statistician was blinded throughout the data assessment process.

### Statistical analysis

Descriptive statistics of all variables were calculated as means and standard deviation. Normality was tested using descriptive statistics, normality tests, and plots (histograms, Q–Q plots, and boxplots). The study variables at T0 and T1 showed normal distribution, while the difference (T1–T0) showed non-normal distribution. Comparisons of the study variables at T0 and T1 between the three groups were performed using one-way ANOVA, while comparisons of the difference (T1–T0) were performed using Kruskal Wallis test. Both tests were followed by multiple pairwise comparisons using Bonferroni adjusted significance level (in case of significant results). Comparisons of the study variables between T0 and T1 within each group were done using paired t-test. The significance level was set at p value < 0.05. Data were analysed using IBM SPSS for Windows (Version 26.0).

### Ethics approval and consent to participate

All the research procedures were performed in accordance with the relevant guidelines and regulations, as stated in the Declaration of Helsinki. The study was approved by the Institutional Review Board of the Faculty of Dentistry, Alexandria University, Alexandria, Egypt (IRB:00010556–IORG:0008839). Manuscript Ethics Committee number 0822-12/2023. Oral assents and written informed consents were obtained from the patients and/or their legal guardians, respectively, prior to the onset of treatment.

## Results

Non-significant differences have been reported between the enrolled participants in each of the three study groups in their baseline data, in terms of age and gender (p > 0.05), as depicted in Table 2. The means and standard deviations for all the evaluated outcomes were calculated at the onset (T0) and at the end of treatment (T1), as well as the differences between them (T1–T0), as displayed in Tables 3 and 4.

**Table 2:** Demographic characteristics of subjects in the three study groups.

		IMA (n = 20)	MB (n = 20)	TB (n = 20)	p value
Age^a^	Mean (SD)	10.31 (1.16)	10.36 (1.04)	10.56 (1.15)	0.76
Gender: n (%)^b^	Male	9 (45%)	11 (55%)	10 (50%)	0.94
	Female	11 (55%)	9 (45%)	10 (50%)	

^a^One-way ANOVA was used.

^b^Chi-square test was used.

**Table 3:** Comparisons of airway area measurements (mm^2^) between the three study groups.

		IMA (n = 20)	MB (n = 20)	TB (n = 20)	p value 1
		Mean (SD)			
NPAA	T0	366.70 (11.57)	372.60 (24.12)	368.20 (10.35)	0.51
	T1	501.85 (23.43) a	442.32 (20.09) b	496.53 (27.96) a	< 0.001*
	Difference	135.15 (19.92) a	69.73 (26.69) b	128.33 (33.58) a	< 0.001*
	p value 2	< 0.001*	< 0.001*	< 0.001*	
OPAA	T0	189.55 (33.14)	173.50 (40.58)	181.75 (33.15)	0.37
	T1	332.60 (18.14) a	230.95 (57.96) b	301.05 (17.04) a	< 0.001*
	Difference	143.05 (33.49) a	57.45 (48.44) b	119.30 (32.71) a	< 0.001*
	p value 2	< 0.001*	0.001*	< 0.001*	
LPAA	T0	289.65 (33.55)	274.35 (28.43)	287.50 (41.93)	0.34
	T1	347.25 (29.95) a	309.25 (13.02) b	337.65 (54.00) a	0.005*
	Difference	57.60 (19.12) a	34.90 (34.76) b	50.15 (25.75) a	0.03*
	p value 2	< 0.001*	< 0.001*	< 0.001*	

Significant values are in [bold].

T0, pre-treatment; T1, post-treatment; SD, standard deviation.

p value 1: Comparisons between the two study groups using one-way ANOVA (for T0 and T1 comparisons), and Kruskal Wallis test (for difference comparisons).

p value 2: Comparisons between T0 and T1 within each group using paired t-test.

a, b: different letters denote statistically significant differences between groups using Bonferroni adjustment.

**Table 4:** Comparisons of lateral cephalometric readings between the three study groups.

		IMA (n = 20)	MB (n = 20)	TB (n = 20)	p value 1
		Mean (SD)			
SNA°	T0	81.10 (1.36)	80.63 (3.05)	81.20 (1.35)	0.65
	T1	80.91 (1.55)	80.40 (3.09)	81.01 (1.55)	0.64
	Difference	− 0.19 (0.44)	− 0.23 (0.70)	− 0.19 (0.45)	0.87
	p value 2	0.07	0.16	0.07	
SNB°	T0	71.89 (3.92)	72.86 (3.53)	71.54 (3.95)	0.53
	T1	75.79 (3.25)	75.36 (3.81)	75.84 (2.58)	0.85
	Difference	3.90 (3.34)	2.50 (3.60)	4.30 (3.32)	0.68
	p value 2	< 0.001*	0.006*	< 0.001*	
ANB°	T0	8.21 (2.47)	7.70 (1.91)	8.66 (2.63)	0.45
	T1	5.22 (1.77)	4.96 (1.86)	5.08 (2.01)	0.91
	Difference	− 3.00 (1.08)	− 2.76 (1.32)	− 3.59 (1.01)	0.06
	p value 2	< 0.001*	< 0.001*	< 0.001*	
Witts appraisal (mm)	T0	7.50 (2.05)	6.40 (1.36)	7.54 (2.08)	0.10
	T1	5.21 (1.97)	4.41 (1.67)	4.99 (1.80)	0.36
	Difference	− 2.30 (0.64)	− 1.99 (1.45)	− 2.56 (0.71)	0.39
	p value 2	< 0.001*	< 0.001*	< 0.001*	
FMA°	T0	32.82 (5.58)	33.57 (3.67)	33.86 (6.48)	0.82
	T1	33.83 (4.86)	32.80 (2.85)	32.98 (2.59)	0.40
	Difference	1.01 (7.78)	− 0.77 (4.18)	− 1.55 (6.12)	0.33
	p value 2	0.57	0.42	0.27	

Significant values are in [bold].

T0, pre-treatment; T1, post-treatment; SD, standard deviation.

p value 1: Comparisons between the two study groups using one-way ANOVA (for T0 and T1 comparisons), and Kruskal Wallis test (for difference comparisons).

p value 2: Comparisons between T0 and T1 within each group using paired t-test.

### Intra-examiner and Inter-examiner reliability

One researcher performed all the measurements at the start. Two weeks later, the same and another calibrated independent examiner repeated the whole measurements on 10 randomly selected x-rays, for analysis of intra and inter-examiner reliability using Intraclass Correlation Coefficient (ICC)^32^. The calculated ICC ranged from 0.82 to 0.96 indicating good to excellent agreement between examiners and across time.

### Measurement of the Sagittal Pharyngeal airway dimension

Changes in the sagittal pharyngeal airway area at the three measured levels NPAA, OPAA, and LPAA are presented in Table 3. A significant increase has been recorded using the IMA, MB, and the TB, between T0 and T1 at all the assessed SPAD levels (p < 0.05). However, when the amount of change recorded at the NPAA, OPAA, and the LPAA was compared between the three study groups, non-significant differences between the IMA and the TB were found, in comparison to the significantly less post-treatment SPAD change elicited in the MB group (p < 0.05).

### Sagittal and vertical skeletal measurements

Changes in both the sagittal and vertical skeletal dimensions in the three study groups, pre- and post-treatment, are described in Table 4. Regarding the sagittal measurements, the IMA, MB, and TB evoked no significant changes in the SNA° values between T0 and T1, with non-significant differences between them as well in comparison (p > 0.05). For the SNB°, a significant increase has been observed between T0 and T1 in the IMA group (3.90° ± 3.34), the MB group (2.50° ± 3.60), and the TB group (4.30° ± 3.32). However, non-significant differences were noted on comparison between all the examined appliances (p > 0.05). ANB° values showed a significant decrease between pre- and post-treatment with IMA, MB, and TB, with respective values of − 3.00° ± 1.08, − 2.76° ± 1.32, and − 3.59° ± 1.01. Nevertheless, non-significant differences between the three test groups were recorded on comparison (p > 0.05). As for Wits appraisal, a similar pattern to that observed with ANB° has been reported, with a statistically significant decrease being documented with all the tested Class II correctives (− 2.30 mm ± 0.64 with IMA, − 1.99 mm ± 1.45 with MB, and − 2.56 mm ± 0.71 with TB), with non-significant differences found between the three test groups (p > 0.05).

For the vertical dimension represented by the FMA° values, non-significant changes have been observed in the three study groups between T0 and T1 (p > 0.05), as well as in the comparisons conducted between all the investigated appliances (p > 0.05).

## Discussion

The present study was conducted to determine the sagittal pharyngeal airway changes concomitant with the use of three Class II correction appliances aiming for mandibular advancement including IMA, MB, and TB. This goes back to the fact that mandibular retrognathism has been proven to be a direct cause of posterior tongue posturing, and the subsequent reduction in the pharyngeal airway capacity^33^. According to the recorded outcomes in this study, the null hypothesis has been rejected, where significant differences between IMA, MB, and TB have been documented regarding the changes in SPAD.

The age of the participants recruited for this study ranged from 9 to 12 years, given that prior research has shown that the impact of functional appliances is optimal at this pre-pubertal stage of rapid growth^34^. This is in agreement with the studies by Baccetti et al.^35^ and Singh et al.^36^, where it has been determined that CVMI stages 3 and 4 represent the ideal treatment timing in dentofacial orthopaedics.

No untreated control group has been incorporated in this retrospective investigation due to ethical concerns, since following up a developing malocclusion without an interceptive corrective action may result in ethical uncertainty. Consequently, no records for a control group were available in the department’s archives for evaluation. Other retrospective studies that assessed airway changes without incorporating a control group include that of Yue et al.^19^, in which the age range of the evaluated sample was relatively close to that included in the current study records, therefore, precedence has been established.

Assessment of pre- and post-treatment SPAD changes was carried out using two-dimensional lateral cephalograms, as they have been the tools of choice for airway assessment in several investigations^26,37,38^. Lateral cephalograms were implemented because of their diminished radiation dose, their cost-effectiveness, in addition to them being readily available as routine radiographs^31^. On a different note, measurement of airway dimensions on lateral cephalograms has been proven to be of high accuracy and reliability^39^. Moreover, a significant correlation has been reported between pharyngeal airway measurements conducted on lateral cephalometric radiographs and those obtained through volumetric measurements performed on three-dimensional (3D) cone-beam computed tomography (CBCT)^40^.

Several researchers have exclusively employed linear metrics in the evaluation of pharyngeal airway parameters^41^. Nonetheless, Aboudara et al.^42^ stated that sagittal airway area measurements were found to exhibit a more significant correspondence to 3D volumetric changes in comparison to linear measurements. Accordingly, sagittal pharyngeal airway area measurements were performed in the present study to compare the effectiveness of IMA, MB, and TB in enhancing the airway dimensions. This has also been the measurement method of choice for SPAD changes in other investigations^26^.

According to the outcomes of the present study, the three tested Class II correction functional appliances, whether the IMA, the MB, or the TB evoked a significant SPAD increase at all the tested levels (NPAA, OPAA, and LPAA) at T1 in comparison to T0. The assessment of airway area changes with IMA has only been studied by Yue et al.^19^, where comparative results to those reported here have been stated, except for a non-significant change being reported at the nasopharyngeal level. They attributed their findings to the resultant forward mandibular movement, which in consequence, elicits a forward pull on the tongue, hyoid bone, and soft tissues of the oropharynx and laryngopharynx, thus expanding the airway area^43^. The non-significant nasopharyngeal increase was justified on their part by the fact that the nasopharynx is surrounded by hard tissue structures, making it less susceptible to environmental changes.

The reported SPAD increase with both MB and TB is in accordance with previous research^7,26,44–47^, which pertains to their effectiveness in forward mandibular posturing and increasing mandibular length^48^, thereupon resulting in an increase in the airway area. For example, Yavan et al.^15^ reported a significant amount of mandibular protraction, together with an increase in the oropharyngeal airway with TB therapy. Moreover, MB has been proven to be effective in the management of Obstructive Sleep Apnea (OSA) in children, through increasing the oropharyngeal airway area^44^. Nevertheless, contradictory findings have been found in the literature where a non-significant influence on the airway dimension using both appliances has been documented^20^. This dissimilarity could be referred to the variation in the measurement methods between different studies, which were sometimes linear, and in others two or three-dimensional volumetric measurements. Thus, it is imperative to consider the employed method of assessment when directly comparing research outcomes, in order to draw definitive conclusions.

Upon comparing the amount of SPAD increase pre- and post-treatment between IMA, MB, and TB, non-significant differences have been reported between both IMA and TB, with the two of them reporting significantly superior results when compared to MB. The reported comparative outcomes achieved using IMA and TB, could be explained by the documented ability of both appliances to encourage anterior and inferior displacement of the hyoid bone, remarking their relative therapeutic effects, despite the reported diversity in controlling the vertical dimension while advancing the mandible using each of them^19^. Moreover, in accordance with our results, Madian and Elfouly^26^ noted a more significant pharyngeal airway area increase with TB in comparison with MB, and their findings were explained by the increased reciprocal force exerted backwards by the TB on the maxilla. This might be added to the exceptional retention of the TB, and its preference by the patients as it does not hinder speech and other daily activities^49,50^. Furthermore, Büyükbayraktar and Camci^20^ reported that the TB was more patient-friendly in contrast to MB especially while sleeping, in addition to the superior retention exhibited with the customized TB that allows precise anterior mandibular positioning, versus the ready-made MB with its flexible material that makes it difficult for the patients to retain their forward mandibular posturing. These reported factors pertain to the superiority of TB in forward mandibular posturing, which in consequence could result in significant airway enhancement as documented in the current study. It is noteworthy to add that the much less wear time with MB, especially during the first month as recommended by the manufacturer^27^, in contrast to the relatively longer wear time with both IMA and TB as mentioned earlier^19,25,26^, could also be a contributing factor to the significantly less impact reported with MB in the present trial.

Regarding the sagittal skeletal changes, it is evident from the present results that all the tested mandibular advancement appliances elicited a remarkable improvement in the sagittal skeletal relationships, given the significant increase in the SNB°, as well as the significant reduction in both ANB° and Wits appraisal. The reported enhancement of intermaxillary relationships using IMA, TB, as well as MB is in accordance with former studies^13,26,51^, where an increase in the mandibular dimensions together with its advancement have been documented, thus improving the sagittal relationships^51^.

Comparisons between the IMA, TB, and MB with regards to the antero-posterior changes showed non-significant differences between the three of them in the amount of SNB°, ANB°, and Wits appraisal at T1. This is in accordance with Madian and Elfouly^26^, who reported non-significant differences in the sagittal changes observed with TB and MB post-treatment. Furthermore, comparative skeletal antero-posterior changes have been noted in a recent study by Lombardo et al.^13^ using both IMA and TB. Their findings were attributed to the similar mechanism of action of both appliances, where inclined planes are incorporated and consequently induce a forced anterior mandibular positioning, while ensuing neuromuscular adaptation^13^.

For the vertical changes, IMA, MB, and TB did not bring about significant changes after treatment, or between the studied groups. This finding has been formerly confirmed in the literature in several studies, whether with TB versus MB^26^, or with IMA versus TB^51^. Contrastingly, it has been reported that IMA surpasses TB in terms of vertical control, producing less clockwise mandibular rotation after advancement^19^. This goes back to the ability of the IMA to intrude the anterior teeth together with mandibular advancement, thus establishing the desirable occlusion without posterior teeth extrusion^52^.

The limitations of the present research encompass the absence of a long-term observation period following the antero-posterior correction, given possible alterations that may occur in the SPAD, which in turn ascertains the need for further prospective clinical trials investigating this area. Additionally, lacking a control group as a benchmark for comparison could be considered a limiting factor. Furthermore, employing two-dimensional lateral cephalometric radiographs is not contemplated as the prime assessment method, in contrast to the three-dimensional methods which also permit volumetric airway evaluation.

## Conclusions

Treatment of skeletal Class II malocclusions with mandibular deficiency using Invisalign Mandibular Advancement (IMA), Myobrace (MB), and Twinblock (TB) functional appliances rendered the following findings:

1. A significant increase in the sagittal pharyngeal airway dimension (SPAD) has been reported using IMA, MB, and TB, with comparative effects using both IMA and TB, both of which were superior to those documented with MB.
2. The three tested appliances elicited a significant improvement in the intermaxillary relationship, evident by a significant increase in SNB°, together with a significant reduction in both ANB° and Wits appraisal, with non-significant differences between the three groups.
3. IMA, MB, and TB did not induce significant changes in the vertical dimension following their use for mandibular advancement.

## Data Availability

The datasets used and/or analyzed during the current study are available from the corresponding author upon reasonable request.

## Abbreviations

SPAD: Sagittal pharyngeal airway dimension
IMA: Invisalign mandibular advancement
TB: Twinblock
MB: Myobrace
PFAs: Prefabricated functional appliances
SD: Standard deviation
CVMI: Cervical vertebral maturational index
NPAA: Nasopharyngeal airway area
OPAA: Oropharyngeal airway area
LPAA: Laryngopharyngeal airway area
H: Harmonium
PNS: Posterior nasal spine
FH: Frankfurt Horizontal plane
C5AI: Antero-inferior point of the 5th cervical vertebra
FMA: Frankfurt-mandibular plane angle
ICC: Intraclass correlation coefficient
3D: Three-dimensional
CBCT: Cone-beam computed tomography
OSA: Obstructive sleep apnea

## References

[1] Alhammadi, M. S., Halboub, E., Fayed, M. S., Labib, A. & El-Saaidi, C. Global distribution of malocclusion traits: A systematic review. *Dental Press J. Orthod.* **23**(40), e1-40.e10 (2018). 10.1590/2177-6709.23.6.40.e1-10.onl PMC6340198 30672991

[2] McNamara, J. A. Jr. Components of class II malocclusion in children 8–10 years of age. *Angle Orthod.* **51**, 177–202 (1981). 7023290 10.1043/0003-3219(1981)051<0177:COCIMI>2.0.CO;2

[3] Proffit, W. R., Fields, H. W. Jr. & Moray, L. J. Prevalence of malocclusion and orthodontic treatment need in the United States: Estimates from the NHANES III survey. *Int. J. Adult Orthodon. Orthognath. Surg.* **13**, 97–106 (1998). 9743642

[4] Indriksone, I. & Jakobsone, G. The upper airway dimensions in different sagittal craniofacial patterns: A systematic review. *Stomatologija* **16**, 109–117 (2014). 25471995

[5] Kirjavainen, M. & Kirjavainen, T. Upper airway dimensions in Class II malocclusion. Effects of headgear treatment. *Angle Orthod.* **77**, 1046–1053 (2007). 18004913 10.2319/081406-332

[6] Nanda, M., Singla, A., Negi, A., Jaj, H. & Mahajan, V. The association between maxillomandibular sagittal relationship and pharyngeal airway passage dimensions. *J. Indian Orthod. Soc.* **46**, 48–52 (2012). 10.1177/0974909820120108

[7] Ghodke, S., Utreja, A. K., Singh, S. P. & Jena, A. K. Effects of twin-block appliance on the anatomy of pharyngeal airway passage (PAP) in class II malocclusion subjects. *Prog. Orthod.* **15**, 68 (2014). 25534004 10.1186/s40510-014-0068-3 PMC4274348

[8] Xiang, M., Hu, B., Liu, Y., Sun, J. & Song, J. Changes in airway dimensions following functional appliances in growing patients with skeletal class II malocclusion: A systematic review and meta-analysis. *Int. J. Pediatr. Otorhinolaryngol.* **97**, 170–180 (2017). 28483230 10.1016/j.ijporl.2017.04.009

[9] Hänggi, M. P., Teuscher, U. M., Roos, M. & Peltomäki, T. A. Long-term changes in pharyngeal airway dimensions following activator-headgear and fixed appliance treatment. *Eur. J. Orthod.* **30**, 598–605 (2008). 18974068 10.1093/ejo/cjn055

[10] Kannan, A., Sathyanarayana, H. P. & Padmanabhan, S. Effect of functional appliances on the airway dimensions in patients with skeletal class II malocclusion: A systematic review. *J. Orthod. Sci.* **6**, 54–64 (2017). 28546958 10.4103/jos.JOS_154_16 PMC5433105

[11] Gill, D., Sharma, A., Naini, F. & Jones, S. The twin block appliance for the correction of Class II malocclusion. *Dent. Update.* **32**, 158–168 (2005). 15881511 10.12968/denu.2005.32.3.158

[12] Gülsoy, B. & Yavan, M. A. Conventional twin-block versus cervical headgear and twin-block combination: Therapeutic effects on the craniofacial structures in growing subjects. *Turk. J. Orthod.* **36**, 149–157 (2023). 37781997 10.4274/TurkJOrthod.2022.2022.84 PMC10548055

[13] Lombardo, E. C. *et al.* Dentoskeletal effects of clear aligner vs twin block-a short-term study of functional appliances. *J. Orofac. Orthop.* **2023**, 89 (2023). 10.1007/s00056-022-00443-1 PMC11358164 36651930

[14] Elfeky, Y. H. & Fayed, M. M. Three-dimensional effects of twin block therapy on pharyngeal airway parameters in Class II malocclusion patients. *J. World Fed. Orthod.* **4**, 114–119 (2015).

[15] Yavan, M. A., Aycan, M., Aysoyler, D. & Essiz, A. Comparison of twin block appliance and Forsus Fatigue Resistant Device therapies on uvuloglossopharyngeal dimensions: A retrospective study. *APOS Trends Orthod.* **11**, 23–31 (2021). 10.25259/APOS_173_2020

[16] Anastasi, G. & Dinnella, A. Myobrace System: A no-braces approach to malocclusion and a myofunctional therapy device. *Orthodontics* **2014**, 5 (2014).

[17] Akin, M., Ucar, F. I., Chousein, C. & Sari, Z. Effects of chincup or facemask therapies on the orofacial airway and hyoid position in Class III subjects. *J. Orofacial Orthop.* **76**, 520 (2015). 10.1007/s00056-015-0315-3 26446505

[18] Oktay, H. & Ulukaya, E. Maxillary protraction appliance effect on the size of the upper airway passage. *Angle Orthod.* **78**, 209–214 (2008). 18251620 10.2319/122806-535.1

[19] Yue, Z. *et al.* Comparison of invisalign mandibular advancement and twin-block on upper airway and hyoid bone position improvements for skeletal class II children: A retrospective study. *BMC Oral Health.* **23**, 661 (2023). 37705022 10.1186/s12903-023-03295-2 PMC10500932

[20] Çoban Büyükbayraktar, Z. & Camcı, H. Dentoalveolar, skeletal, pharyngeal airway, cervical posture, hyoid bone position, and soft palate changes with Myobrace and Twin-block: A retrospective study. *BMC Oral Health.* **23**, 53 (2023). 36717838 10.1186/s12903-023-02773-x PMC9887833

[21] Petrie, A. & Sabin, C. *Medical Statistics at a Glance* 3rd edn. (Blackwell, 2009).

[22] Hassel, B. & Farman, A. G. Skeletal maturation evaluation using cervical vertebrae. *Am. J. Orthod. Dentofacial Orthop.* **107**, 58–66 (1995). 7817962 10.1016/S0889-5406(95)70157-5

[23] Savoldi, F. *et al.* Reliability of lateral cephalometric radiographs in the assessment of the upper airway in children: A retrospective study. *Angle Orthod.* **90**, 47–55 (2020). 31403838 10.2319/022119-131.1 PMC8087055

[24] Malkoc, S., Usumez, S., Nur, M. & Donaghy, C. E. Reproducibility of airway dimensions and tongue and hyoid positions on lateral cephalograms. *Am. J. Orthod. Dentofacial Orthop.* **128**, 513–516 (2005). 16214635 10.1016/j.ajodo.2005.05.001

[25] Buyukcavus, M. H. & Kale, B. Skeletal and dental effects of twin-block appliances in patients treated with or without expansion. *Turk. J. Orthod.* **34**, 155–162 (2021). 35110185 10.5152/TurkJOrthod.2021.20103 PMC8939253

[26] Madian, A. M. & Elfouly, D. Cephalometric changes in pharyngeal airway dimensions after functional treatment with twin block versus myobrace appliances in developing skeletal class II patients: A randomized clinical trial. *BMC Oral Health.* **23**, 998 (2023). 38093237 10.1186/s12903-023-03701-9 PMC10720117

[27] Achmad, H. & Auliya, N. Management of malocclusion in children using myobrace appliance: A systematic review. *F1000Res* **13**, 53 (2024). 10.12688/f1000research.51879.1

[28] Johnson, J. S. *et al.* A comparative evaluation of the dentoskeletal treatment effects using twin block appliance and myobrace system on class II division I malocclusion. *Int. J. Clin. Pediatr. Dent.* **14**, S10–S17 (2021). 35082460 10.5005/jp-journals-10005-2013 PMC8754264

[29] Clark, W. J. Twin block functional therapy. Applications in dentofacial orthopaedics. *Orthodontics* **1995**, 856 (1995).

[30] Ramirez-Yañez, G., Sidlauskas, A., Junior, E. & Fluter, J. Dimensional changes in dental arches after treatment with a prefabricated functional appliance. *J. Clin. Pediatr. Dent.* **31**, 279–283 (2007). 19161066 10.17796/jcpd.31.4.d7p31201572n72h2

[31] Chand, K., Jacob, S. & Charles, A. Assesment of changes in the sagittal pharyngeal airway dimensions post twin-block therapy using polar planimeter. *SRM J. Res. Dent. Sci.* **8**, 51–57 (2017). 10.4103/srmjrds.srmjrds_79_16

[32] Koo, T. K. & Li, M. Y. A guideline of selecting and reporting intraclass correlation coefficients for reliability research. *J. Chiropr. Med.* **15**, 155–163 (2016). 27330520 10.1016/j.jcm.2016.02.012 PMC4913118

[33] Jena, A. K., Singh, S. P. & Utreja, A. K. Sagittal mandibular development effects on the dimensions of the awake pharyngeal airway passage. *Angle Orthod.* **80**, 1061–1067 (2010). 20677955 10.2319/030210-125.1 PMC8929504

[34] Jena, A. K., Duggal, R. & Parkash, H. Skeletal and dentoalveolar effects of Twin-block and bionator appliances in the treatment of Class II malocclusion: A comparative study. *Am. J. Orthod. Dentofacial Orthop.* **130**, 594–602 (2006). 17110256 10.1016/j.ajodo.2005.02.025

[35] Baccetti, T., Franchi, L., Toth, L. R. & McNamara, J. A. Jr. Treatment timing for Twin-block therapy. *Am. J. Orthod. Dentofacial Orthop.* **118**, 159–170 (2000). 10935956 10.1067/mod.2000.105571

[36] Singh, S. *et al.* Timing of myofunctional appliance therapy. *J. Clin. Pediatr. Dent.* **35**, 233–240 (2010). 21417131 10.17796/jcpd.35.2.9572h13218806871

[37] Kim, J. E. *et al.* Effects of the long-term use of maxillary protraction facemasks with skeletal anchorage on pharyngeal airway dimensions in growing patients with cleft lip and palate. *Korean J. Orthod.* **50**, 238–248 (2020). 32632043 10.4041/kjod.2020.50.4.238 PMC7369382

[38] Baloş Tuncer, B., Ulusoy, Ç., Tuncer, C., Türköz, Ç. & Kale Varlik, S. Effects of reverse headgear on pharyngeal airway in patients with different vertical craniofacial features. *Braz. Oral Res.* **29**, 1–8 (2015). 10.1590/1807-3107BOR-2015.vol29.0057 25992786

[39] Restrepo, C., Santamaría, A., Peláez, S. & Tapias, A. Oropharyngeal airway dimensions after treatment with functional appliances in class II retrognathic children. *J. Oral Rehabil.* **38**, 588–594 (2011). 21294763 10.1111/j.1365-2842.2011.02199.x

[40] Riley, R., Powell, N. & Guilleminault, C. Cephalometric roentgenograms and computerized tomographic scans in obstructive sleep apnea. *Sleep* **9**, 514–515 (1986). 3809865 10.1093/sleep/9.4.514

[41] Jeans, W. D., Fernando, D. C., Maw, A. R. & Leighton, B. C. A longitudinal study of the growth of the nasopharynx and its contents in normal children. *Br. J. Radiol.* **54**, 117–121 (1981). 7459548 10.1259/0007-1285-54-638-117

[42] Aboudara, C. *et al.* Comparison of airway space with conventional lateral headfilms and 3-dimensional reconstruction from cone-beam computed tomography. *Am. J. Orthod. Dentofacial Orthop.* **135**, 468–479 (2009). 19361733 10.1016/j.ajodo.2007.04.043

[43] Tsuiki, S., Lowe, A. A., Almeida, F. R., Kawahata, N. & Fleetham, J. A. Effects of mandibular advancement on airway curvature and obstructive sleep apnoea severity. *Eur. Respir. J.* **23**, 263–268 (2024). 10.1183/09031936.04.00094304 14979501

[44] Ahn, E. S., Kim, A. H., Shim, Y. S. & An, S. Y. Oropharyngeal airway three-dimensional changes after treatment with myobrace in class II retrognathic children. *Iran. J. Public Health.* **46**, 265–267 (2017). 28451565 PMC5402788

[45] Yıldırım, E. & Karaçay, Ş. Volumetric evaluation of pharyngeal airway after functional therapy. *Scanning.* **2021**, 6694992 (2021). 33680278 10.1155/2021/6694992 PMC7906813

[46] Göymen, M., Mourad, D. & Güleç, A. Evaluation of airway measurements in class II patients following functional treatment. *Turk. J. Orthod.* **32**, 6–10 (2019). 30944893 10.5152/TurkJOrthod.2019.18050 PMC6436910

[47] Mohamed, R. N., Basha, S. & Al-Thomali, Y. Changes in upper airway dimensions following orthodontic treatment of skeletal Class II malocclusion with twin block appliance: A systematic review. *Turk. J. Orthod.* **33**, 59 (2020). 32284900 10.5152/TurkJOrthod.2020.19028 PMC7138231

[48] Elhamouly, Y., El-Housseiny, A. A., Ismail, H. A. & Habashy, L. M. E. Myofunctional trainer versus twin block in developing class II division I malocclusion: A randomized comparative clinical trial. *Dent. J.* **8**, 44 (2020). 10.3390/dj8020044 PMC7345969 32392835

[49] Mohammed, H., Čirgić, E., Rizk, M. Z. & Vandevska-Radunovic, V. Effectiveness of prefabricated myofunctional appliances in the treatment of Class II division 1 malocclusion: A systematic review. *Eur. J. Orthod.* **42**, 125–134 (2020). 31329848 10.1093/ejo/cjz025

[50] Ferreira, F. Novel approaches for class II malocclusion treatment using myofunctional orthodontics therapy: A systematic review. *Int. J. Dent. Oral Sci.* **4**, 503–507 (2017).

[51] Caruso, S. *et al.* Mandibular advancement with clear aligners in the treatment of skeletal Class II. A retrospective controlled study. *Eur. J. Paediatr. Dent.* **22**, 26–30 (2021). 33719479 10.23804/ejpd.2021.22.01.05

[52] Blackham, S. S. A study of short-term skeletal, dental, and soft tissue effects of Class II malocclusions treated with InvisalignⓇ with Mandibular Advancement Feature or Twin Block appliance compared with historical controls: Doctoral dissertation, University of British Columbia (2020).

